# Sex differences in children’s cognitive functions and phthalates exposure: a meta-analysis

**DOI:** 10.1038/s41390-023-02672-5

**Published:** 2023-06-01

**Authors:** Yu-Chi Liao, Yi-Jia Xu, Jing-Kai Chen, Hathaichon Boonhat, Bei-Yi Su, Yi-Chun Lin, Ro-Ting Lin

**Affiliations:** 1 Department of Psychology, College of Medical and Health Science, Asia University, Taichung, Taiwan; 2 Center for Prevention and Treatment of Internet Addiction, Asia University, Taichung, Taiwan; 3 Clinical Psychology Center, Asia University Hospital, Taichung, Taiwan; 4 Graduate Institute of Public Health, College of Public Health, China Medical University, Taichung, Taiwan; 5 Department of Psychology, Chung-Shan Medical University, Taichung, Taiwan; 6 Room of Clinical Psychology, Chung Shan Medical University Hospital, Taichung, Taiwan; 7 Department of Chinese Medicine, China Medical University Hospital, Taichung, Taiwan; 8 Department of Occupational Safety and Health, College of Public Health, China Medical University, Taichung, Taiwan

## Abstract

**Background:**

Phthalates exposure might affect children’s intelligence development. This study aimed to determine (1) whether sex and age affect cognitive function and (2) whether sex differences in cognitive performance are wider with higher phthalate concentrations.

**Methods:**

Data were collected from PubMed (1998–2022), PROQUEST (1997–2022), and SpringerLink (1995–2022). The study followed the PRISMA process. The included articles were followed by PECO framework. The GRADE applied to assess the certainty of evidence. Of 2422 articles obtained, nine were selected using inclusion criteria. The random-effects model was used to estimate the pooled effects.

**Results:**

Our meta-regression indicated a significant difference between sex differences with age at phthalate concentration assessment (*β* = −0.25; 95% CI = −0.47, −0.03) and MEHP concentration (*β* = −0.20; 95% CI = −0.37, −0.03).

**Conclusions:**

The limitation of the current article is it only provides information on intelligence level rather than other aspects of cognitive function. Thus, the sequelae of phthalate exposure on attention and executive function are still unclear. Our analysis shows significant difference between sex differences in cognitive function scores associated with age at phthalate concentration assessment. Girls might be more resilient in cognitive function at a younger age or during lower concentrations of phthalates metabolites.

**Impact:**

- This is the first meta-analysis to evaluate the pooled estimates of sex differences in objective cognitive functions among children with phthalate exposure.
- The female might be a protective factor when exposed to toxic plasticizers while the concentration is low.
- This study captures the possible role of sex in cognitive functioning and plasticizer exposure through a meta-analysis of children’s sex, cognitive scores, and plasticizer exposure.

## Introduction

Phthalates are common endocrine-disrupting chemicals in our daily lives, found in plastics ranging from bottles to flooring. After entering the body, most phthalates are excreted in urine and feces within 24 h.^1^ Nevertheless, repeated exposure to high levels of phthalates during pregnancy, breastfeeding, and infancy may impair neurodevelopment,^2^ including intelligence. Intelligence broadly refers to an individual’s ability to combine logical reasoning, comprehension, expression, language, and learning, profoundly affecting lifelong learning.^3^

As both prenatal and postnatal exposure to phthalates affects the development of cognitive function, an increasing number of epidemiological studies have assessed the potential association; however, the conclusions are varied because of several methodological discrepancies.^4,5^ The three main factors causing discrepancies include diverse types of phthalates, different indicators of cognition level (e.g., varying from using the Behavioral Assessment System for Children, Second Edition [BASC-2] to measure behavioral and emotional functioning to using the Bayley Scales of Infant and Toddler Development Version II [BSID-II] to measure mental and psychomotor developmental functions^4^), and potential biological differences in subjects (e.g., age and sex). Therefore, until a recent meta-analysis was published by Radke et al. in 2020,^6^ most review articles were unable to conduct a meta-analysis to quantify the effect of phthalate exposure on cognition.^4,7,8^ This meta-analysis used five to eight articles to assess the associations between five phthalates (i.e., DEHP, DBP, DIBP, BBP, and DEP) and two types of cognitive function in children (i.e., infant mental and psychomotor development).^6^ While most results revealed slight and non-significant inverse associations, there was a moderate and significant inverse association between BBP and psychomotor development in girls,^6^ indicating that sex may moderate the extent to which phthalates affect cognition.^9^

Before 3 years of age, girls usually have an earlier developmental timetable than boys, so sex differences in cognition level may exist.^10^ Jankowska reviewed the role of sex in neurodevelopment on plasticizer exposure and found that phthalates are essential factors determining children’s cognitive, psychomotor, behavioral, and emotional development.^8^ However, their findings had many confounding variables; the outcomes they included were merged with subjective and objective measures, and the cognitive function results were contaminated. In addition, they had no conclusions about cognitive functions after plasticizer exposure. It is still unclear whether sex differences in cognition levels persist when children reach a certain age.

Standard intelligence tests used to assess research and clinical measures could be differentiated by age, below and above the age of three. The most common measurement of intelligence for children below 3 years of age is the BSID versions I, II, and III.^11^ The most common measurement for children above 3 years of age is the Wechsler Intelligence Scale (WIS),^12,13^ but some studies used the Intelligence and Development Scales (IDS) as an alternative.^14^ Each measure compares the norms for each age and sex group, and we should consider whether these differences vary with phthalate exposure. Therefore, this meta-analysis investigated (1) whether sex and age affect cognitive performance and (2) whether sex differences in cognitive performance are wider with higher phthalate concentrations.

## Methods

We collected data from three databases: PubMed (1998–2022), PROQUEST (1997–2022), and SpringerLink (1995–2022). We used EndNoteX9 for importation and articles management. Restrictions were not put on the publication date or language of the publications, and the last search was conducted on December 23, 2022. The search keywords were categorized into three categories, each of which contained all three components: (1) phthalates; (2) child, preschool, infant, or teenager; and (3) cognitive function or intelligence.

This meta-analysis was conducted based on the Preferred Reporting Items for Systematic Reviews and Meta-Analyses (PRISMA) process,^15^ reported in Supplementary Table 1. We followed the population, exposure, comparator, and outcome (PECO) framework to review and included articles in the meta-analysis. First, the population was defined as children aged 0–16 years. Second, the exposure and comparator were defined as male children (hereafter referred to as boys) vs. female children (hereafter referred to as girls) in different age groups and high vs. low phthalate exposure. Third, the outcome was lower cognitive performance, measured using a standardized intelligence score. The intelligence score currently measured using relevant ability scales is based on country and age norms. The standard deviation score was 15. We excluded articles in the first screening stage that were (1) non-human clinical trials, (2) non-child articles, (3) non-English language articles, (4) without cognitive assessment, and (5) not including the plasticizer metabolites data. In the final screening stages, we excluded the articles that (1) missing data, (2) overlapping population comprised duplicate data (i.e., different articles using the same data source; if this was the case, the article was selected for analysis with complete data), (3) cognitive assessment raw data was not provided. Two independent reviewers screened the relevant articles. Each article was evaluated for suitability for inclusion in the PRISMA process, as well as a keyword search of the article. If a discrepancy occurred, a third reviewer participated in the discussion until a consensus was reached.

We used Microsoft Excel for data curation and Comprehensive Meta-Analysis 3.0 and Stata version 16.1 (StataCorp, TX) for statistical analysis. Data records and descriptive statistics, such as participant distribution, number of participants, data sources, and measurement of cognitive function tools, were recorded using Microsoft Excel. A comprehensive meta-analysis was used to compare sex and cognitive function differences across the study and draw forest plots. Meta-regression was used to evaluate sex differences in cognitive function as a factor affecting phthalates metabolite content. Mean differences between the sexes were weighted by the standard deviation of the mean and the number of participants. Funnel plots and Egger’s tests were used to evaluate potential publication bias.^16^

We used the National Toxicology Program Office of Health Assessment and Translation (NTP/OHAT) approach for evidence integration to assess the risk of bias and GRADE to grade the certainty of evidence.^17^ We used I-squared (*I*^*2*^) and tau-squared (τ^2^) to investigate heterogeneity across selected studies between-study variance in the random-effects meta-analysis,^16^ respectively. *I*^*2*^ ranged from 0–25%, 25–50%, 50–75%, and >75%, representing no heterogeneity, low heterogeneity, moderate heterogeneity, and high heterogeneity, respectively.^18^

## Results

### Study selection and characteristics

**Fig. 1:**
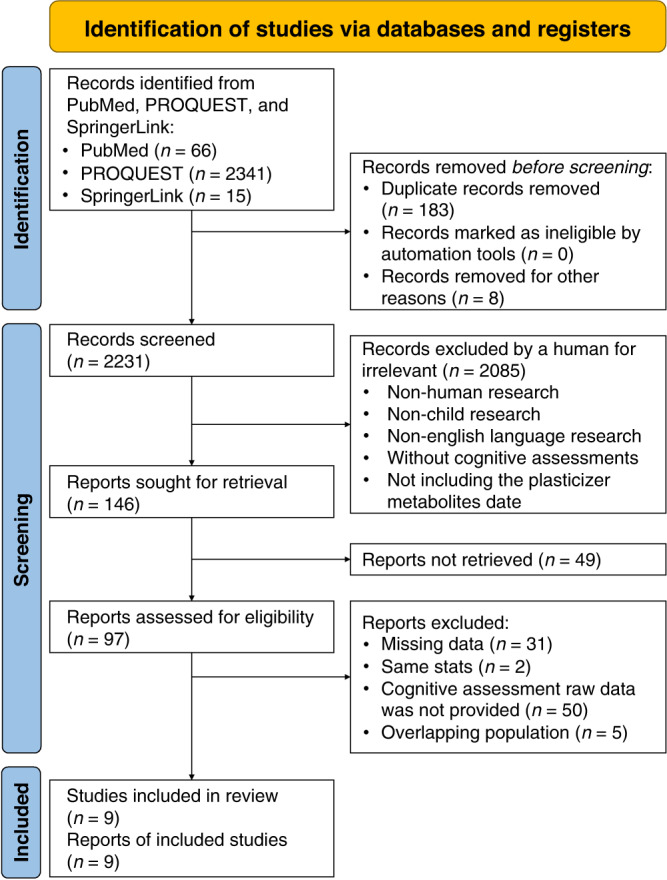
Process of selecting articles. n number of articles.

Figure 1 shows the selected articles following the PRISMA process. The number of included articles in the initial stage was 2422. After removing 183 duplicate articles and eight book references, the number became 2231. In the next stage, 146 articles were included after applying the inclusion and exclusion criteria mentioned above. In the final screening stage, 31 articles lacked complete data, two had the same data, 50 did not provide complete cognitive assessment data, and five overlapping populations were excluded. Nine articles met the inclusion criteria. Eight of the 97 articles included in the review showed inconsistency in results between two independent reviewers, which were resolved through arbitration by a third reviewer. The inter-rater reliability of the selected articles was 0.92.

These nine articles included twelve study groups: one each from Poland, Sweden, Italy, Denmark, and Slovakia; two each from South Korea, and the United States; and three each from Taiwan. Participants were recruited from hospitals, environmental health centers, obstetrics and gynecology clinics, and child psychiatric clinics. Concerning cognitive instruments, three articles used Bayley, and nine used WIS. Regarding the timing of phthalate concentration assessment, five articles (five study groups) measured prenatal samples (mother’s urine), and four articles (seven study groups) measured postrenal samples (children’s urine). The characteristics of the nine articles included in the final analysis are summarized in Table 1.

**Table 1:** Essential characteristics and review of the articles included in the meta-analysis.

Authors	Country	Tools and tool name	Timing of phthalate assessment	Study period, number of subjects, and age at intelligence assessment age (year)	Phthalates	Major findings
Cho et al. 2010	South Korea	Tool: WIS Tool name: KEDI-WIS	Postnatal	Study period: 2008 Number of subjects: 344 boys and 323 girls Age at intelligence assessment: 9.05 y	MEHP, MEOHP, and MBP	(*n* = 667); There is a negative association between increasing MEHP phthalate concentrations and the sum of DEHP metabolite concentrations and the Wechsler Intelligence Scale for children’s vocabulary score; however, among girls, there is no significant association between these variables.
Kim et al. 2011	South Korea	Tool: Bayley (MDI) Tool name: Bayley BSID-II	Prenatal	Study period: 2006–2009 Number of subjects: 235 boys and 225 girls Age at intelligence assessment: 0.50 y	MEOHP, MEHHP, MBP, and ∑DEHP	(*n* = 460); The results suggest that prenatal exposure to phthalates may be inversely associated with MDI and PDI in infants, especially males, at 6 months of age.
Whyatt et al. 2012	The United States	Tool: Bayley (MDI) Tool name: Bayley BSID-II	Prenatal	Study period: 1999–2006 Number of subjects: 157 boys and 140 girls Age at intelligence assessment: 0.70 y	MEHP, MEOHP, MEHHP, MECPP, ∑DEHP, MBzP, MiBP, and MnBP	(*n* = 297); Results suggest that prenatal exposure to phthalates may adversely affect a child’s mental, motor, and behavioral development during preschool.
Doherty et al. 2017	The United States	Tool: Bayley (MDI) Tool name: Bayley BSID-II	Prenatal	Study period: 1998–2002 Number of subjects: 131 boys and 116 girls Age at intelligence assessment: 2.67 y	MEHP, MEOHP, MEHHP, MECPP, ∑DEHP, MBzP, MiBP, MnBP, MEP, and MCPP	(*n* = 247); There are no associations between phthalate metabolite concentrations and performance on the MDI or PDI in boys and girls combined. However, among girls, metabolite associations may be inversely associated with MDI and PDI. Conversely, we observed multiple weakly positive associations among boys.
Huang et al. 2017	Taiwan	Tool: WIS Tool name: WPPSI-R	Postnatal	Study period: 2012–2013 Number of subjects: 67 boys and 41 girls Age at intelligence assessment: 4.60 y	MEHP, MEOHP, MEHHP, ∑DEHP, MiBP, MnBP, and ∑DBP	(*n* = 204); The findings suggested that the current exposure to phthalates for school-aged children aged ≥6– < 12 years was significantly and negatively associated with VCI performance. Maternal IQ was slightly positively associated with neurodevelopment for participants aged ≥3– < 12 years. The data revealed that the current exposure to DEHP and DBP might affect the children’s nervous system, which might be shown in their language learning or expression ability.
		Tool: WIS Tool name: WISC-IV	Postnatal	Study period: 2012–2013 Number of subjects: 56 boys and 40 girls Age at intelligence assessment: 7.60 y		
Jankowska et al. 2019	Poland	Tool: WIS Tool name: IDS (Fluid intelligence)	Prenatal	Study period: 2007–2014 Number of subjects: 57 boys and 72 girls Age at intelligence assessment: 7 y	MEHP, ∑DEHP, MBzP, MiBP, MnBP, and MEP	(*n* = 123); The results suggested that the IDS analyses focused on a child’s cognitive and psychomotor development are inconclusive. There was a significantly negative association between phthalates and fluid intelligence and cognition during early childhood, while positive associations have been found during the prenatal period.
Tsai et al. 2020	Taiwan	Tool: WIS Tool name: WISC-IV	Postnatal	Study period: Not reported Number of subjects: 42 boys and 26 girls Age at intelligence assessment: boys = 8.70 y and girls = 9.20 y	Nor reported	(*n* = 68); According to the results of this article, the possibility of an adverse impact of phthalate on neurodevelopment may exist, particularly in boys.
Rosolen et al. 2022	Italy	Tool: WIS Tool name: WISC-IV	Postnatal	Study period: 2014–2016 Number of subjects: 150 boys and 150 girls Age at intelligence assessment: 7 y	MBzP, MiBP, MnBP, and MEP	(*n* = 900); In NAC-II, a direct association between FSIQ and biomarkers of DEHP was found. In contrast, in OCC, the relationship between biomarkers of DEHP and FSIQ tended to be inverse but imprecise (*p* ≥ 0.10). No associations were found in the PCB cohort. In conclusion, these results do not provide evidence for an association between concurrent phthalate exposure and IQ in children.
	Denmark	Tool: WIS Tool name: WISC-V	Postnatal	Study period: 2018–2019 Number of subjects: 165 boys and 135 girls Age at intelligence assessment: 7 y		
	Slovakia	Tool: WIS Tool name: WISC-III	Postnatal	Study period: 2014–2017 Number of subjects: 133 boys and 167 girls Age at intelligence assessment: 11 y		
Gennings et al. 2022	Sweden	Tool: WIS Tool name: WISC-IV	Prenatal	Study period: 2007–2017 Number of subjects: 332 boys and 346 girls Age at intelligence assessment: 7 y	MEP, MBP, MBzP, and ∑DEHP	(*n* = 678); The findings suggested that early prenatal exposure to phthalates is associated with lower levels of cognitive functioning at age seven. This adverse association is particularly stronger in boys.

### Meta-analysis: sex comparison for cognitive function

**Fig. 2:**
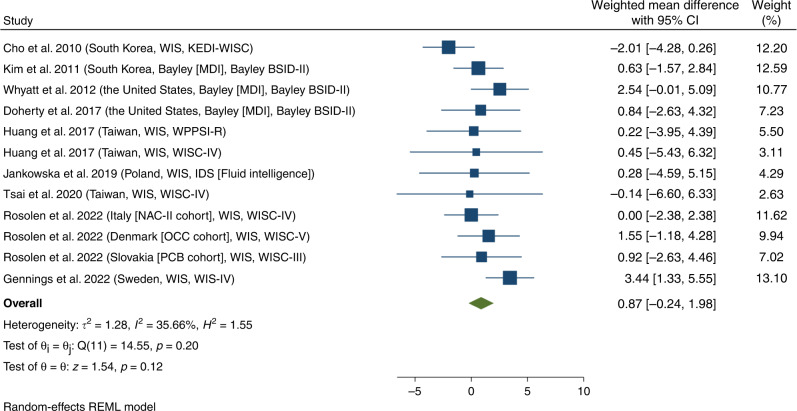
Forest plot of studies on the weighted mean difference of intelligence scores between boys and girls with phthalates exposures. BSID-II Bayley Scales of Infant and Toddler Development Version II, *H*^*2*^ h-squared, IDS Intelligence and Development Scales, *I*^*2*^ I-squared, *p* *p*-value, KEDI-WISC Korean Educational Development Institute-Wechsler Intelligence Scale for Children, *τ*^*2*^ tau-squared, WIS Wechsler Intelligence Scale.

**Fig. 3:**
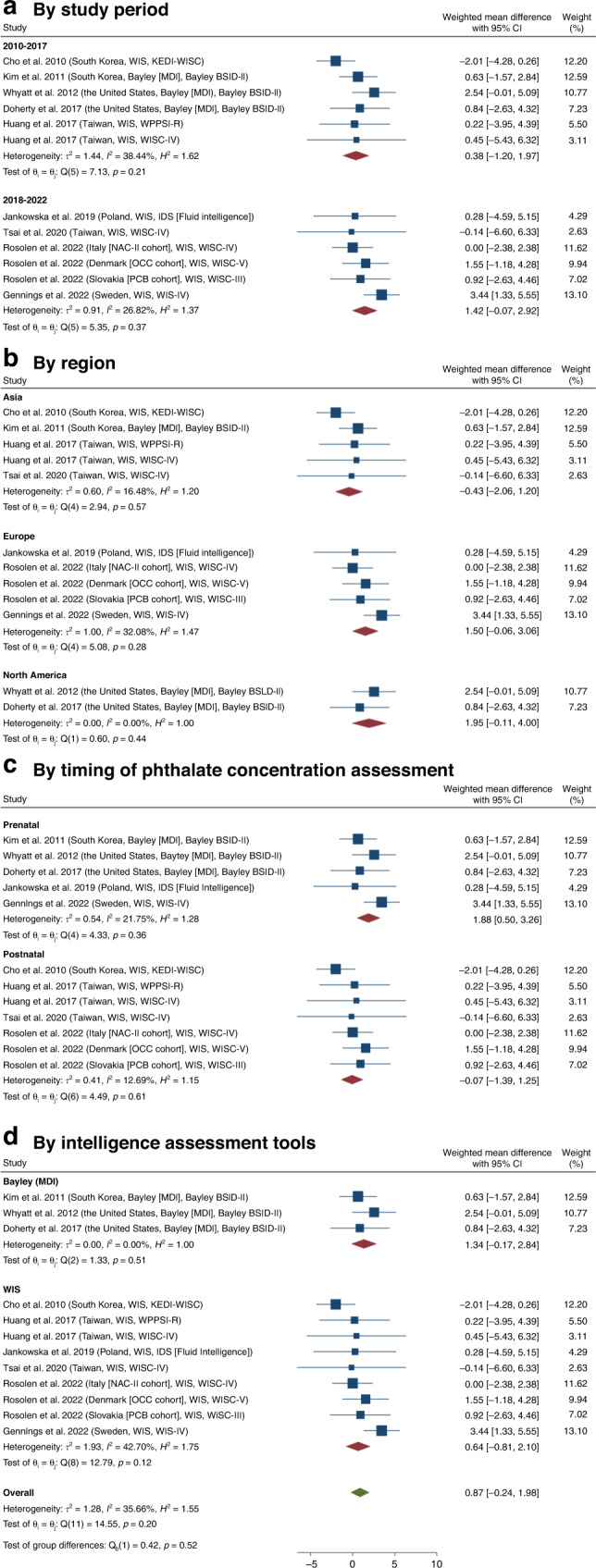
Subgroup analysis of studies on the weighted mean difference of intelligence scores between boys and girls with phthalates exposures. **a** By study period, **b** by region, **c** by the timing of phthalate concentration assessment, and **d** by intelligence assessment tools. BSID-II Bayley Scales of Infant and Toddler Development Version II, *H*^*2*^ *h*-squared, IDS Intelligence and Development Scales, *I*^*2*^ I-squared, *p* *p*-value, KEDI-WISC Korean Educational Development Institute-Wechsler Intelligence Scale for Children, *τ*^*2*^ tau-squared, WIS Wechsler Intelligence Scale.

The significant difference in cognitive scores between the sexes was not detected (weighted mean difference = 0.87; 95% CI = −0.24, 1.98; *p* = 0.20; *I*^*2*^ = 35.66%) (Fig. 2). Additionally, we performed subgroup analyses by study period, region, the timing of phthalate concentration assessment, and intelligence assessment tools (Table 2; Fig. 3a–d).

**Table 2:** Subgroup analysis between plasticizer exposure by different characteristics.

Characteristics	Number of study groups	Weighted mean difference (95% CI)	*I*^*2*^ (in %)	*p*-value	Tau^2^	Group difference (*p*-value of *Q* test)
Overall	12	0.87 (−0.24, 1.98)	35.66	0.20	1.28	
Study period						0.35
2010–2017	6	0.38 (−1.20, 1.97)	38.44	0.21	1.44	
2018–2022	6	1.42 (−0.07, 2.92)	26.82	0.37	0.91	
Region						0.13
Asia	5	−0.43 (−2.06, 1.20)	16.48	0.57	0.60	
Europe	5	1.50 (−0.06, 3.06)	32.08	0.28	1.00	
North America	2	1.95 (−0.11, 4.00)	0.00	0.44	0.00	
Timing of phthalate concentration assessment						0.05
Prenatal	5	1.88 (0.50, 3.26)	21.75	0.36	0.54	
Postnatal	7	−0.07 (−1.39, 1.25)	12.69	0.61	0.41	
Intelligence assessment tools						0.52
Bayley	3	1.34 (−0.17, 2.84)	0.00	0.51	0.00	
WIS + IDS	9	0.64 (−0.81, 2.10)	42.70	0.12	1.93

### Meta-regression and sex role in cognitive functions

**Fig. 4:**
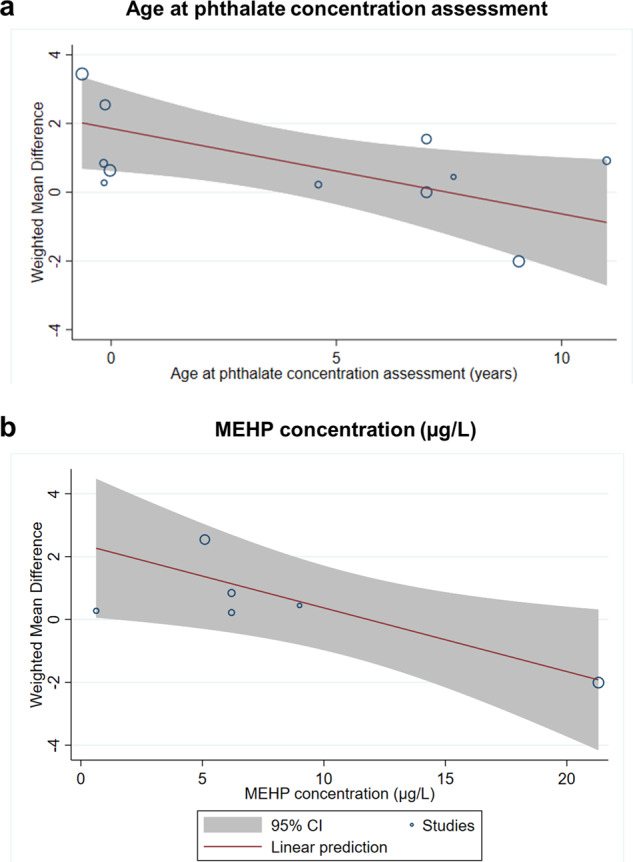
Meta-regression. Meta-regression of the weighted mean difference in (**a**) age at phthalate concentration assessment and (**b**) MEHP concentration (μg/L).

Figure 4 reveals bubble plots of sex difference in cognitive function scores associated with age at phthalate concentration assessment (Fig. 4a; $\beta$ = −0.25; 95% CI = −0.47, −0.03; *p* = 0.03), and MEHP concentration (Fig. 4b; $\beta$ = −0.20; 95% CI = −0.37, −0.03; *p* = 0.02). Supplementary Fig. 1A–I shows insignificant results on MEOHP ($\beta$ = −0.01; 95% CI = −0.22, 0.21; *p* = 0.96), MEHHP ($\beta$ = −0.00; 95% CI = −0.11, 0.13; *p* = 0.97), ∑DEHP ($\beta$ = 0.02; 95% CI = −0.03, 0.07; *p* = 0.37), MBzP ($\beta$ = 0.00; 95% CI = −0.06, 0.06; *p* = 0.96), MiBP ($\beta$ = −0.01; 95% CI = −0.05, 0.03; *p* = 0.50), MnBP ($\beta$ = 0.01; 95% CI = −0.07, 0.10; *p* = 0.74), MBP ($\beta$ = 0.03; 95% CI = −0.14, 0.21; *p* = 0.71), MEP ($\beta$ = −0.00; 95% CI = −0.03, 0.02; *p* = 0.85), and age at intelligence assessment ($\beta$ = −0.14; 95% CI = −0.48, 0.19; *p* = 0.40).

Further correlation analysis revealed that MEHP concentration was also associated with age at phthalate concentration assessment (both prenatal and postnatal; *r* = 0.71; *p* < 0.05; see Supplementary Fig. 2A) and age at intelligence assessment (*r* = 0.40; *p* < 0.05; see Supplementary Fig. 2B). However, the correlation coefficient between “MEHP concentration” and “age at phthalate concentration assessment (both prenatal and postnatal)” was higher. This finding suggests that the difference was more significant at younger ages, probably due to the lower concentration of age at the phthalate concentration assessment. In addition, both correlations showed that the higher the age, the higher the plasticizer concentration measured. Our findings suggest that the protective effect of age may only exist at young ages or low concentrations of plasticizers.

### Publication bias

**Fig. 5:**
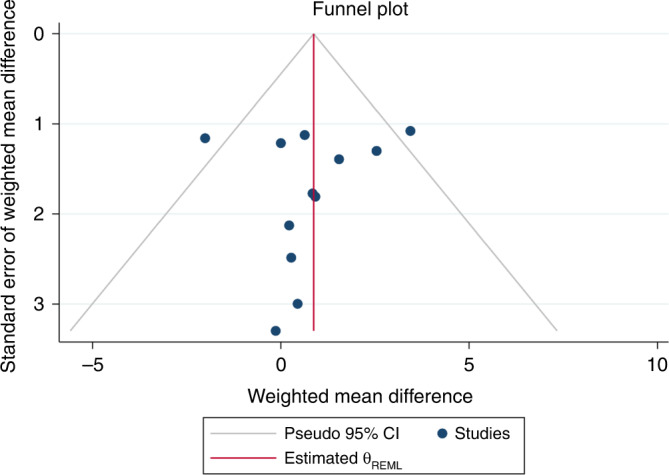
Funnel plot. Funnel plot for the weighted mean difference from the articles included in the meta-analysis of twelve study groups.

A symmetric inverted funnel shape indicated a low risk of publication bias among these nine articles with twelve study groups, as shown in Fig. 5 ($\beta$ = −0.47; 95% CI = 0.59, 1.49; *p* = 0.66).

### Risk of bias

The risk of bias assessments for studies investigating the association between plasticizer exposure and intelligence function are summarized in Table 3, and the details of each assessment are provided in Supplementary Table 2. The NTP/OHAT Risk of Bias tool focuses on confounding and detection biases, including exposure and outcome assessment. We assessed the risk of bias among the nine articles. For confounding bias, confounders such as parents’ smoking habits and education, household income, and lower socioeconomic status were not adjusted throughout the four articles. Thus, three articles were rated as having a “probably high risk of bias.”^19,20^ For exposure detection bias, none of the articles was rated as “probably low risk of bias.” However, for outcome detection bias, two articles were rated as “probably high risk of bias,” which provided insufficient information to justify the assessment.^21^ For exclusion bias, five articles that did not specify exclusion bias were rated as “probably high risk of bias.”^20–23^ Four articles that failed to report conflicts of interest or funding support were rated as “probably high risk.”^20–23^ For selective reporting bias, article was rated as “probably low risk of bias” due to a lack of significant and consistent evidence of inverse associations between three groups of cohort study exposures and FSIQ. In summary, articles were categorized as Tier 1 (*n* = 5) or Tier 2 (*n* = 4) for risk of bias, indicating a “plausible bias that raises some doubt about the results.”^17^

**Table 3:** The assessment of the risk of bias of plasticizers articles the NTP/PHAT risk of bias rating tool for children’s plasticizer cohort study.

Bias domain		Cho et al., 2010.	Doherty et al., 2017	Whyatt et al., 2012	Huang et al., 2017	Jankowska et al., 2019	Kim et al., 2011	Tsai et al., 2020	Rosolen et al., 2022	Gennings et al., 2022
++	Definitively low risk of bias									
+	Probably low risk of bias									
-	Probably high risk of bias									
--	Definitively high risk of bias									
Confounding bias										
1. Did the study design or analysis account for important confounding and modifying variables? (Key domain)		+	++	+	+	-	+	-	-	+
Detection bias										
1. Can we be confident in the exposure characterization? (Key domain)		+	++	+	+	+	++	++	++	++
2. Can we be confident in the outcome assessment? (Key domain)		-	+	+	+	+	+	++	-	+
Selection bias										
1. Did selection of study participants result in appropriate comparison groups?		+	++	+	+	+	++	++	+	+
Attrition/ Exclusion bias										
1. Where outcome data complete without attrition or exclusion from analysis?		-	++	+	-	-	-	+	-	++
Selective reporting bias										
1. Were all measured outcome reported?		+	+	+	+	+	+	++	+	-
Other bias										
1. Conflict of interest		-	++	-	+	-	-	+	+	++
Summary tier category		T2	T1	T1	T1	T2	T1	T2	T2	T1

### Certainty of evidence

A summary of the findings regarding the certainty of the evidence is provided in Table 4. Regarding the risk of bias rating for studies investigating plasticizers, the overall risk of bias was “not serious.” The inconsistency of the certainty assessment indicated low heterogeneity in the meta-analyses of the studies (*I*^*2*^ = 35.66%). However, the explanation for this heterogeneity could reveal some inconsistencies, such as differences in study design, population age, prenatal or postnatal exposure, and statistical methods. The respective category of the inconsistency of certainty assessment was “serious.” The indirectness and imprecision certainty assessment indicated a category of “not serious” because the outcomes of our study demonstrated that the evidence answers directly to population cognitive functions living near plasticizers, and the CI of the pooled analysis was narrow (−0.24 to 1.98). Funnel plots (Fig. 5) exhibited symmetrical patterns, and weighted mean difference did not yield evidence of publication bias, indicating an undetectable publication bias. In summary, the inherent risk of bias made our rating for the certainty of evidence from low to very low for the association between sex differences in cognitive function and plasticizer exposure.

**Table 4:** Summary of finding evidence.

Certainty assessment							№ of patients		Effect	Certainty
№ of studies	Study design	Risk of bias	Inconsistency	Indirectness	Imprecision	Other considerations	Boys exposed to plasticizers	Girls exposed to plasticizers	Absolute (95% CI)	
9	observational studies	serious^a^	serious	not serious	not serious	none	2033	2087	beta 0.47 lower (0.59 higher to 1.49 higher)	⨁◯◯◯ very low

*CI* confidence interval, *GRADE* Grading of Recommendations Assessment, Development, and Evaluation.

^a^Confounding bias was founded in 3 out of 9 articles.

## Discussion

To the best of our knowledge, this is the first meta-analysis to evaluate the pooled estimates of sex differences in objective cognitive functions among children with phthalate exposure. We analyzed the data of twelve study groups from Poland, Sweden, Italy, Denmark, Slovakia, South Korea, Taiwan, and the United States. There was no significant difference in cognitive scores between sexes. However, our meta-regression of the weighted mean difference shows a significant difference between sex differences in cognitive function scores and age at phthalate concentration assessment. In other word, the younger the age of the child, the greater cognitive and difference in phthalate concentration between sexes. Another meta-regression of the weighted mean difference shows significant between sex differences in cognitive function scores and MEHP concentration. Specifically, the timing of phthalate concentration assessment and MEHP concentration from 6 reports were significantly associated with sex differences by a factor of 0.2 ($\beta$ = −0.20; 95% CI = −0.37, −0.03; *p* = 0.02). The estimated sex differences in intelligence scores were higher among children with prenatal phthalate exposure and lower MEHP concentrations. Suggesting that when the concentration of MEHP is lower, sex plays as a protecting factor, making the difference in average IQ between males and females larger. Also, the difference in intelligence decreased by 0.02–0.03 points if the timing of phthalate measurement started 1 year later, and MEHP concentration (geometric mean) increased 1 (µg/L).

We observed a higher score for cognitive function in girls than in boys when phthalate exposure was at a younger age than at an older age. This finding suggests that girls have greater physical resilience when exposed to phthalates at a young age.^24^ A previous systematic review has shown that being female would act as a protective factor that could modulate the effects of substance residues on a child’s cognitive function, including bisphenol A and plasticizers.^9^ The reason prenatal or early exposure to phthalates had more adverse effects on neurodevelopment in males than in females might be that plasticizers could disrupt the development of androgen-dependent structures by inhibiting fetal testicular testosterone biosynthesis.^25^ Past studies further showed that in animal or human research, substance exposure would affect male cognitive function and not females and even cause behavioral problems.^22,26,27^

However, does phthalate do more harm to neurodevelopment in males than in females? Research on cognitive and intellectual development has discussed sex differences in the early stages of life; for example, girls had better intelligence than boys when they were 2 to 7 years old, and it was reported that girls had better processing speed from ages 4 to 7.^28^ Additionally, male toddlers were observed to be less tractable and manageable than girls, which could cause more injury or less parenting at early ages.^29^ However, as children grow up, these differences would dismiss.^30,31^ We found that female works as physical resilience at a younger early age.

Our correlation results showed a strong relationship between age (age at intelligence assessment and age at phthalate concentration assessment) and the concentration of phthalates metabolites; as the child grows up, more phthalates metabolites are detected. This phenomenon indicates that phthalates metabolites would affect the child strongly when exposed to more plastic products in their daily lives.

### Limitations

This study has several strengths. First, cognitive function was measured by a professional psychologist following a standardization process to ensure objectivity. Moreover, the cognitive scores of the included articles proved that both boys and girls had normative cognitive scores (the mean cognitive score of norms was 100). The study objectively answers the review published by ref. ^8^ The included articles encompass different continents, such as Asia, North America, and Europe, to increase holistically.

However, our study had some limitations. First, the included articles measured intelligence levels. There was a lack of assessing other aspects of cognitive function, such as executive function and attention. Although recent intelligence measurements claimed that they could measure executive function or attention,^32^ these neuropsychological functions did not fully report in plasticizer exposure articles. These cognitive functions involve different concepts and do not always have similar outcomes.^33^ Thus, the sequelae of phthalate exposure on attention and executive function are still unclear. Future studies should include a comparison group (control group) and different objective cognitive function measurements. Additionally, phthalates were measured at different times (e.g., environmental measurements only and maternal or child subjects). Jankowska et al. reported different results in a prenatal and postnatal sampling study.^20^ It shows that prenatal (third trimester) and postnatal (at 2 years of age) measurements indicated that the prenatal phthalate metabolites were lower than those measured postnatally. Further studies have shown that different sequelae range between prenatal and postnatal exposures.^20,34^ Last and all, only observational studies were included in the analysis; we could only use the norm’s mean and standard deviation to check if the intelligence score of studies showed would be within or without the norm’s range. Nor were the studies comparing the intelligence score with their country/area demographic similar norms. In addition, a comparison of the exposure sample with the control sample is lacking. We suggest future studies should include a control group to examine the sequelae of exposure, even with artificial intelligence techniques, to find the critical neurodevelopmental disorder after exposure to a plasticizer.^35^

## Conclusion

We suggest that girls had resilient cognitive function in cognitive scores and lower concentrations of phthalate metabolites compared with the boys at the same exposure level. Therefore, the female might be a protective factor when exposed to toxic plasticizers while the concentration is low. Moreover, when the child grows up, more phthalate metabolites are detected, suggesting that phthalate metabolites might affect the child strongly as they are exposed to more plastic products in their daily lives.

### Supplementary information

Supplementary Materials

## Data Availability

Data from the primary studies included in this meta-analysis are available upon request from the corresponding author. The data consist of were collected from PubMed, PROQUEST, and SpringerLink between 1995–2022. Interested researchers must obtain permission from the original study authors and adhere to any applicable ethical guidelines. Researchers must also agree to cite the original studies and seek permission before using the data for any purpose other than that of the current meta-analysis.

## References

[1] Frederiksen H, Skakkebaek NE, Andersson AM (2007). Metabolism of phthalates in humans. Mol. Nutr. Food Res..

[2] Braun JM (2017). Early-life exposure to EDCs: role in childhood obesity and neurodevelopment. Nat. Rev. Endocrinol..

[3] Hunt, J. M. *Intelligence and Experience* (Ronald), (1961).

[4] Ejaredar M, Nyanza EC, Ten Eycke K, Dewey D (2015). Phthalate exposure and childrens neurodevelopment: a systematic review. Environ. Res..

[5] Martínez-Martínez MI, Alegre-Martínez A, Cauli O (2021). Prenatal exposure to phthalates and its effects upon cognitive and motor functions: a systematic review. Toxicology.

[6] Radke EG, Braun JM, Nachman RM, Cooper GS (2020). Phthalate exposure and neurodevelopment: a systematic review and meta-analysis of human epidemiological evidence. Environ. Int..

[7] Zhang Q, Chen XZ, Huang X, Wang M, Wu J (2019). The association between prenatal exposure to phthalates and cognition and neurobehavior of children-evidence from birth cohorts. Neurotoxicology.

[8] Jankowska A, Nazareth L, Kaleta D, Polanska K (2021). Review of the existing evidence for sex-specific relationships between prenatal phthalate exposure and children’s neurodevelopment. Int. J. Environ. Res. Public Health.

[9] Palanza P (2021). Sex-biased impact of endocrine disrupting chemicals on behavioral development and vulnerability to disease: of mice and children. Neurosci. Biobehav. Rev..

[10] Halpern, D. F. *Sex Differences in Cognitive Abilities* (Psychology press, 2000).

[11] Lowe JR, Erickson SJ, Schrader R, Duncan AF (2012). Comparison of the Bayley Ii Mental Developmental Index and the Bayley Iii cognitive scale: are we measuring the same thing?. Acta Paediatr..

[12] Wechsler, D. *Wechsler Intelligence Scale for Children* 4th edn (Wisc-Iv, 2003).

[13] Wechsler, D. Wechsler Preschool and Primary Scale of Intelligence—Fourth Edition. *The Psychological Corporation**San Antonio, TX* (2012).

[14] Grob, A., Meyer, C. & Hagmann-von Arx, P. *Intelligence and Development Scales (Ids)* (Hans Huber, 2009). 10.1024/1422-4917/a000148 22161941

[15] Page MJ (2021). The prisma 2020 statement: an updated guideline for reporting systematic reviews. BMJ.

[16] Higgins, J P. T. (eds). *Cochrane Handbook for Systematic Reviews of Interventions version 6.3 (updated February 2022)* (Cochrane). Retrieved on July 14, 2022 from https://training.cochrane.org/handbook (2022).

[17] Office of Health Assessment and Translation(OHAT) Division of the National Toxicology Program. *Handbook for Conducting a Literature-Based Health Assessment Using Ohat Approach for Systematic Review and Evidence Integration* (National Institute of Environmental Health Sciences). Retrieved on July 14, 2022 from https://ntp.niehs.nih.gov/ntp/ohat/pubs/handbookmarch2019_508.pdf (2019).

[18] Higgins JP, Thompson SG, Deeks JJ, Altman DG (2003). Measuring inconsistency in meta-analyses. BMJ.

[19] Tsai CS (2020). Phthalates, para-hydroxybenzoic acids, bisphenol-A, and gonadal hormones’ effects on susceptibility to attention-deficit/hyperactivity disorder. Toxics.

[20] Jankowska A (2019). Prenatal and early postnatal phthalate exposure and child neurodevelopment at age of 7 years - polish mother and child cohort. Environ. Res..

[21] Cho SC (2010). Relationship between environmental phthalate exposure and the intelligence of school-age children. Environ. Health Perspect..

[22] Kim Y (2011). Prenatal exposure to phthalates and infant development at 6 months: prospective mothers and children’s environmental health (Moceh) study. Environ. Health Perspect..

[23] Whyatt RM (2012). Maternal prenatal urinary phthalate metabolite concentrations and child mental, psychomotor, and behavioral development at 3 years of age. Environ. Health Perspect..

[24] Bruneau, M. & Reinhorn, A. *Proc. 8th US National Conference on Earthquake Engineering*. 18–22.

[25] Yen TH, Lin-Tan DT, Lin JL (2011). Food safety involving ingestion of foods and beverages prepared with phthalate-plasticizer-containing clouding agents. J. Formos. Med. Assoc..

[26] Dai Y, Yang Y, Xu X, Hu Y (2015). Effects of uterine and lactational exposure to Di-(2-Ethylhexyl) phthalate on spatial memory and NMDA receptor of hippocampus in mice. Horm. Behav..

[27] Lai T-J (2002). A cohort study of behavioral problems and intelligence in children with high prenatal polychlorinated biphenyl exposure. Arch. Gen. Psychiatry.

[28] Palejwala MH, Fine JG (2015). Gender differences in latent cognitive abilities in children aged 2 to 7. Intelligence.

[29] Matheny AP (1986). Injuries among toddlers: contributions from child, mother, and family1. J. Pediatr. Psychol..

[30] Reynolds MR, Hajovsky DB, Caemmerer JM (2022). The sexes do not differ in general intelligence, but they do in some specifics. Intelligence.

[31] Saggino A (2014). Null sex differences in general intelligence among elderly. Pers. Individ Differ..

[32] Montoya-Arenas DA, Aguirre-Acevedo DC, Díaz Soto CM, Pineda Salazar DA (2018). Executive functions and high intellectual capacity in school-age: completely overlap?. Int J. Psychol. Res..

[33] Ardila A, Pineda D, Rosselli M (2000). Correlation between intelligence test scores and executive function measures. Arch. Clin. Neuropsychol..

[34] Polańska K, Ligocka D, Sobala W, Hanke W (2016). Effect of environmental phthalate exposure on pregnancy duration and birth outcomes. Int J. Occup. Med. Environ. Health.

[35] Allahyari E, Roustaei N (2022). Applying artificial neural-network model to predict psychiatric symptoms. BioMedicine.

