# Supplementary material for: Sex differences in children’s cognitive functions and phthalates exposure: a meta-analysis
Source: Pediatr Res. 2023 Jun 1;94(5):1609–18. doi: 10.1038/s41390-023-02672-5 (PMC10624603; doi:10.1038/s41390-023-02672-5)
Supplement: Supplementary file 1 — Supplementary Materials [file 41390_2023_2672_MOESM1_ESM.pdf]

Supplementary file

Table 1. PRISMA checklist

Section and Topic	Item #	Checklist item	Location where item is reported
**TITLE**			
Title	1	Identify the report as a meta-analysis.	1
**ABSTRACT**			
Abstract	2	See the PRISMA 2020 for Abstracts checklist.	3
**INTRODUCTION**			
Rationale	3	Describe the rationale for the review in the context of existing knowledge.	4–5
Objectives	4	Provide an explicit statement of the objective(s) or question(s) the review addresses.	5
**METHODS**			
Eligibility criteria	5	Specify the inclusion and exclusion criteria for the review and how studies were grouped for the syntheses.	5–7
Information sources	6	Specify all databases, registers, websites, organisations, reference lists and other sources searched or consulted to identify studies. Specify the date when each source was last searched or consulted.	5–6
Search strategy	7	Present the full search strategies for all databases, registers and websites, including any filters and limits used.	5–6
Selection process	8	Specify the methods used to decide whether a study met the inclusion criteria of the review, including how many reviewers screened each record and each report retrieved, whether they worked independently, and if applicable, details of automation tools used in the process.	6
Data collection process	9	Specify the methods used to collect data from reports, including how many reviewers collected data from each report, whether they worked independently, any processes for obtaining or confirming data from study investigators, and if applicable, details of automation tools used in the process.	6
Data items	10a	List and define all outcomes for which data were sought. Specify whether all results that were compatible with each outcome domain in each study were sought (e.g. for all measures, time points, analyses), and if not, the methods used to decide which results to collect.	6
	10b	List and define all other variables for which data were sought (e.g. participant and intervention characteristics, funding sources). Describe any assumptions made about any missing or unclear information.	6
Study risk of bias assessment	11	Specify the methods used to assess risk of bias in the included studies, including details of the tool(s) used, how many reviewers assessed each study and whether they worked independently, and if applicable, details of automation tools used in the process.	7
Effect measures	12	Specify for each outcome the effect measure(s) (e.g. risk ratio, mean difference) used in the synthesis or presentation of results.	6–7
Synthesis methods	13a	Describe the processes used to decide which studies were eligible for each synthesis (e.g. tabulating the study intervention characteristics and comparing against the planned groups for each synthesis (item #5)).	6–7
	13b	Describe any methods required to prepare the data for presentation or synthesis, such as handling of missing summary statistics, or data conversions.	6–7
	13c	Describe any methods used to tabulate or visually display results of individual studies and syntheses.	6–7
	13d	Describe any methods used to synthesize results and provide a rationale for the choice(s). If meta-analysis was performed, describe the model(s), method(s) to identify the presence and extent of statistical heterogeneity, and software package(s) used.	6–7
	13e	Describe any methods used to explore possible causes of heterogeneity among study results (e.g. subgroup analysis, meta-regression).	7
	13f	Describe any sensitivity analyses conducted to assess robustness of the synthesized results.	7
Reporting bias assessment	14	Describe any methods used to assess risk of bias due to missing results in a synthesis (arising from reporting biases).	7
Certainty assessment	15	Describe any methods used to assess certainty (or confidence) in the body of evidence for an outcome.	7
**RESULTS**			
Study selection	16a	Describe the results of the search and selection process, from the number of records identified in the search to the number of studies included in the review, ideally using a flow diagram.	7
	16b	Cite studies that might appear to meet the inclusion criteria, but which were excluded, and explain why they were excluded.	7
Study characteristics	17	Cite each included study and present its characteristics.	8
Risk of bias in studies	18	Present assessments of risk of bias for each included study.	9–10
Results of individual studies	19	For all outcomes, present, for each study: (a) summary statistics for each group (where appropriate) and (b) an effect estimate and its precision (e.g. confidence/credible interval), ideally using structured tables or plots.	8
Results of syntheses	20a	For each synthesis, briefly summarise the characteristics and risk of bias among contributing studies.	8–10
	20b	Present results of all statistical syntheses conducted. If meta-analysis was done, present for each the summary estimate and its precision (e.g. confidence/credible interval) and measures of statistical heterogeneity. If comparing groups, describe the direction of the effect.	9–10
	20c	Present results of all investigations of possible causes of heterogeneity among study results.	9–10
	20d	Present results of all sensitivity analyses conducted to assess the robustness of the synthesized results.	9–10
Reporting biases	21	Present assessments of risk of bias due to missing results (arising from reporting biases) for each synthesis assessed.	9–10
Certainty of evidence	22	Present assessments of certainty (or confidence) in the body of evidence for each outcome assessed.	10
**DISCUSSION**			
Discussion	23a	Provide a general interpretation of the results in the context of other evidence.	11
	23b	Discuss any limitations of the evidence included in the review.	13
	23c	Discuss any limitations of the review processes used.	13
	23d	Discuss implications of the results for practice, policy, and future research.	12, 14
**OTHER INFORMATION**			
Registration and protocol	24a	Provide registration information for the review, including register name and registration number, or state that the review was not registered.	The review was not registered
	24b	Indicate where the review protocol can be accessed, or state that a protocol was not prepared.	A protocol was not prepared
	24c	Describe and explain any amendments to information provided at registration or in the protocol.	n/a
Support	25	Describe sources of financial or non-financial support for the review, and the role of the funders or sponsors in the review.	20
Competing interests	26	Declare any competing interests of review authors.	20
Availability of data, code and other materials	27	Report which of the following are publicly available and where they can be found: template data collection forms; data extracted from included studies; data used for all analyses; analytic code; any other materials used in the review.	15

From: Page MJ, McKenzie JE, Bossuyt PM, Boutron I, Hoffmann TC, Mulrow CD, et al. The PRISMA 2020 statement: an updated guideline for reporting systematic reviews. BMJ 2021;372:n71. doi: 10.1136/bmj.n71

Table 2. Risk of bias of studies based on the NTP/OHAT risk of bias tool (NTP/OHAT, 2019).

Domain of risk of bias\Study	Cho et al., 2010,	Doherty et al., 2017	Whyatt et al., 2012	Huang et al., 2017	Jankowska et al., 2019a	Kim et al., 2011	Tsai et al., 2020	Rosolen et al., 2022	Gennings et al., 2022
**Selection bias**<br><br>Did selection of study participants result in appropriate comparison groups?	Probably low<br><br>Participants from five South Korean cities were recruited to investigate the relationship between urinary phthalate metabolite concentrations and children's intellectual functioning while controlling for maternal characteristics such as IQ score and cigarette smoking.	Definitively low<br><br>Participants were recruited from New York City between 1998-2002, with eligible maternal participants having a singleton pregnancy, attending their first prenatal care visit before 26 weeks of gestation, being free of serious chronic conditions or pregnancy complications, and not reporting consumption of more than two alcoholic beverages per day or use of illicit drugs.	Probably low<br><br>The study involved pregnant women who delivered between 1999 and 2006; researchers controlled for maternal age, smoking, race, illicit drug use, gestational age, education level, household income, and region of residence while measuring phthalate metabolite concentrations in spot urine samples collected during pregnancy.	Probably low<br><br>Study participants were recruited from individuals receiving consultation services at 128 Taiwanese hospitals and subsequently transferred to specialty clinics at three participating hospitals following the 2011 plasticizer contamination report, hailing from Taipei, Taichung, and Kaohsiung; demographic data, including age, sex, residence, maternal education level, socioeconomic status, health status, and environmental exposure factors, was collected via questionnaires.	Probably high<br><br>The participants were recruited within the same time frame using the same inclusion and exclusion criteria and were of similar age and health status. However, there is insufficient information provided about the subject sources.	Low<br><br>The pregnant participants who were above 18 years were recruited from 3 cities in Korea Seoul, Cheonan, and Ulsan. The children from 2006 through 2009 were examined at 6 months of age.	Low<br><br>The comparison groups are recruited from the same area. Researcher recruited children who were treated in the outpatient Department of Child Psychiatry at Kaohsiung Chang Gung Children's Hospital in Taiwan.	Probably low<br><br>Participants were recruited using the same ascertainment method, time frame, and inclusion and exclusion criteria across four European geographical regions (north, east, south, and west), representing the European population and accounting for confounding variables such as sex, urban or rural residence, and socioeconomic status.	Probably low<br><br>The comparison groups are recruited from the same area. The selected area is regarded as representative of the general Swedish population.
**Confounding bias**<br><br>Did the study design or analysis account for important confounding and modifying variables?	Probably low<br><br>Phthalates were measured in children aged 9 during April and October 2008 and 2009, with adjustments made for demographic and developmental covariates, although indirect evidence suggested potential confounders were not adjusted for in the final analyses.	Definitively low<br><br>In an additional sensitivity analysis, trained examiners administered the Bayley Scales for Infant Development-II to examine analytical approaches and validity, while the study focused on primiparous women, collected detailed sociodemographic data, and adjusted for important confounding factors such as metabolite concentrations, age, and sex.	Probably Low<br><br>In addition to sex and age, standardization by other covariates such as socioeconomic status (SES), ethnicity, or income. This indicated that the authors were adjusted for important confounding factors like SES, age, and sex.	Probably low<br><br>While the current study may not have accounted for all factors affecting child neurodevelopment, it included critical factors like maternal IQ, cigarette smoking, and education, yet indirect evidence suggested that potential confounders were not adjusted for in the final analyses.	Low<br><br>The study used SDQ logistic regression covariates to adjust for the child's sex, child's age at examination, birth weight, SES, maternal educational level, prenatal and childhood tobacco smoke exposure, breastfeeding duration, and maternal BMI. There is direct evidence that appropriate adjustment considerations were made for primary covariates and confounders in the final analyses.	Probably low<br><br>The covariates were based on self-reported information given during the study interview. The variables were categorical and modeled using categories, including birth weight, breastfeeding time, yearly household income, and maternal education.	Probably high<br><br>Researchers employed the general linear model to assess differences in endocrine-disrupting chemicals and gonadal hormones between ADHD and control groups, adjusting for age, urinary creatinine levels, and specific gravity as covariates, although indirect evidence suggests that primary covariate and known confounder distribution differences between groups were not adequately adjusted for in the final analyses.	Probably high<br><br>Based on information obtained from the questionnaires applied in the different aligned studies, variables corresponded across the studies and data/variables. Potential confounders in neurodevelopment to the three cohorts were the following: the highest education level of the household of the child, body mass index (BMI) z-score, and sex of the child, was modeling.	Probably low<br><br>The variable was adjusted for child sex, maternal nutrition index, energy, age at birth, pre-pregnancy weight, education level, maternal IQ, and smoking status. These variables are considered appropriate adjustments. The study did not adjust for potential confounding factors, such as prematurity and birth weight.
**Attrition/ Exclusion bias**<br><br>Were outcome data complete without attrition or exclusion from analysis?	Probably high<br><br>There is indirect evidence of incomplete outcome data, as inconsistencies exist between the number of recruited and outcome participants without an explanation for participant attrition.	Definitively low<br><br>Clearly explain there are two participants were excluded due to extremely dilute urine (creatinine < 10 mg/dL). There is no evidence of exclusion bias.	Probably Low<br><br>A consistent number of participants reported on the outcome results. There were no other significant differences between boys and girls.	Probably high<br><br>There is indirect evidence that the exclusion of subjects from analyses was not adequately addressed. Only the source of the subject was mentioned.	Probably high<br><br>There is indirect evidence of incomplete outcomes of data. The inconsistent number of participants was provided on the recruited and outcome participant without explaining the reasons for participant attrition in the outcome report.	Probably high<br><br>The article only partially explains the number of participants and the reasons for inclusion and exclusion, but the number of partial exclusions remains unspecified.	Probably low<br><br>From the methods, eligibility criteria for cases were all incident; the researcher excluded those patients who had a comorbid autism spectrum disorder, intellectual disability, psychotic disorders, major depressive disorder, bipolar disorder, or neurological disorders.	Probably high<br><br>No specified exclusion biases. No state about the loss to follow-up of participants.	Low<br><br>There is no evidence of exclusion bias, as the method description indicates that data for mother-child pairs with complete data were selected from the Swedish Environmental Longitudinal Mother and Child, Asthma and Allergy (SELMA) pregnancy cohort.
**Detection bias**<br>1. Can we be confident in the exposure characterization?	Probably low<br><br>Phthalates were measured using urine sampling, while maternal and children's cognitive functions were assessed separately, with the children being individually evaluated by a licensed clinical psychologist with 20 years of experience.	Definitively low<br><br>There is direct evidence that exposure was consistently assessed. The phthalates were measured by urine sampling of creatinine concentration.	Probably low<br><br>The study collected spot urine samples from maternal participants during the third trimester of pregnancy, with each analytical run including calibration standards, reagent blanks, and quality control samples, while specific gravity correction for urinary dilution, as recommended for phthalates, was measured using a handheld refractometer.	Probably low<br><br>Indirect evidence suggests that acceptable methods were used to assess outcomes, including urinary sampling methodology with high-performance liquid chromatography coupled with tandem mass spectrometry (HPLC-MS/MS) and limits of detection (LOD), indicating consistent exposure assessment through well-established, direct measurement techniques.	Probably low<br><br>Data from the prospective Polish Mother and Child Cohort Study (REPRO_PL), established in 2007, was approved by the Nofer Institute of Occupational Medicine's Ethical Committee and included informed consent from pregnant women and children's parents for scheduled cohort phases, providing direct evidence of consistent exposure assessment.	Probably low<br><br>The article describes in detail the geographic distribution and number of participants. There is direct evidence that exposure was consistently assessed.	Low<br><br>The phthalates were measured using urine sampling. The children's cognitive function was measured separately and measured by a senior psychiatrist and child psychologist.	Low<br><br>The phthalates were measured using urine sampling. In the three cohorts, the neurodevelopment of children was assessed by trained psychologists using the WISC test.	Low<br><br>The phthalates were measured using blood and urine sampling. The children's cognitive function was measured by a trained psychiatrist.
2. Can we be confident in the outcome assessment?	Probably high<br><br>There is indirect evidence that the cross-sectional design cannot rule out the possibility of misclassification between exposure and outcome.	Probably low<br><br>Indirect evidence suggests that acceptable methods were used to assess outcomes; however, the single spot urine sample collected during one trimester may not represent exposure throughout pregnancy due to phthalates' short biological half-lives, and while measurement error in phthalate metabolite concentration is suspected to be non-differential, it is still relevant to important qualities such as child's neurologic development, phthalate exposure, and confounding variables.	Probably low<br><br>The phthalates were measured using urine sampling for the same length of time in all study groups. The outcome assessor was assessed using an acceptable method.	Probably low<br><br>The phthalates were analyzed with an LC-ESE-MS/MS (Agilent 1200/ API4000). The subsequence procedure used well-established methods.	Probably low<br><br>The phthalates were measured using urine sampling for the same length of time in all study groups. The outcome assessor was assessed using acceptable methods.	Probably low<br><br>Phthalate metabolite measurements followed the Centers for Disease Control and Prevention (CDC) Laboratory Procedure Manual using urine samples, while cognitive function assessment was conducted by trained examiners with high interrater reliability (kappa value > 0.8) in quiet rooms for 30 to 45 minutes.	Probably low<br><br>There is indirect evidence that the cross-sectional design cannot rule out the possibility of misclassification between exposure and outcome.	Probably high<br><br>The data were obtained from European Human Biomonitoring Initiative (HBM4EU) aligned studies. There is indirect evidence that the outcome was assessed using acceptable methods.	Probably low<br><br>From the text, phthalates and cognitive data were obtained from the SELMA pregnancy cohort. There is direct evidence that the outcome was assessed using well-established methods.
**Selective reporting bias**<br>Were all measured outcomes reported?	Probably low<br><br>The outcome has been reported in methods and abstract with 95%CI. This outcome provides sufficient detail to be included in the meta-analysis.	Probably low<br><br>The outcome has been reported in methods and abstract with 95%CI. This outcome provides sufficient detail to be included in the meta-analysis.	Probably Low<br><br>Metabolites were detected in 84-100% of urine samples, and as expected, the four DEHP metabolite concentrations, adjusted for specific gravity, showed high correlation (Spearman's *r* values .68-.97), leading to their conversion to molecular weights and summed (ΣDEHP); outcomes were reported in methods and abstract with 95%CI, providing sufficient detail for inclusion in the meta-analysis.	Probably low<br><br>The research protocol was approved by the Research Ethics Committee of the National Health Research Institutes (No. EC1000903) and the collaborating hospitals.<br>The outcome has been reported in the methods and abstract. This outcome provides sufficient detail to be included in the meta-analysis.	Probably low<br><br>The outcome has been reported in methods and abstract with 95%CI. This outcome provides sufficient detail to be included in the meta-analysis.	Probably high<br><br>The outcome has been reported in methods and abstract with 95%CI. This outcome provides sufficient detail to be included in the meta-analysis.	Probably low<br><br>The outcome has been reported in the abstract and method, following the protocol of the study. This outcome provides sufficient detail to be included in the meta-analysis.	Probably low<br><br>The results of this study protocol evaluation lack significant and consistent evidence of inverse associations between this group's exposures and FSIQ. All outcomes have been reported crudely and adjusted β and 95% CI of all linear regression models.	Probably high<br><br>Data were analyzed using random splits (e.g., 40% for weight estimation and 60% for hypothesis testing) and potentially multiple random splits to address generalizability, with the ensemble's strength lying in the increased identification of concerning agents, albeit at the cost of forgoing model parsimony.
**Other bias**<br>Conflict of interest	Probably high<br><br>No specified.	Definitively low<br><br>This study was supported by National Institute for Environmental Health Sciences and Environmental Protection Agency Children's Center. The authors declare there is no conflict of interest.	Probably high<br><br>No specified.	Probably low<br><br>The authors declare that no competing interests exist.	Probably high<br><br>No specified.	Probably high<br><br>No specified	Low<br><br>This research was funded by Chang Gung Memorial Research Projects, with the authors declaring no conflicts of interest and the sponsors having no involvement in the study's design, execution, interpretation, or writing.	Probably low<br><br>Informed consent was obtained from all participant's caregivers involved in the study. The authors declare no conflict of interest.	Low<br><br>This research was supported by the National Institutes of Health for biostatistical methods development and the European Union's Horizon 2020 Research and Innovation Program for analytical samples; participants provided written consent, and the study was approved by the Ethical Review Board in Uppsala, Sweden, and the authors declared no conflicts of interest.

Abbreviations: ADHD, attention deficit hyperactivity disorder; ASL, local health authority of Novara; CI, confidence interval; CO, carbon monoxide; ICD, International Classification of Disease; IDS, Intelligence and Development Scales; NH_3, ammonia; O_3, ozone; OPIS, Opis EnviMan ComVisioner and EnviMan Reporter; OR, odds ratio; PM, particulate matter; RIF, Rapid Inquiry Facility; RR, relative risk; SDQ, Strengths and Difficulties Questionnaire; SIR, standardized incidence rate; SO_2, sulfur dioxide; UTM, Universal Transverse Mercator; VOCs, volatile organic compounds.

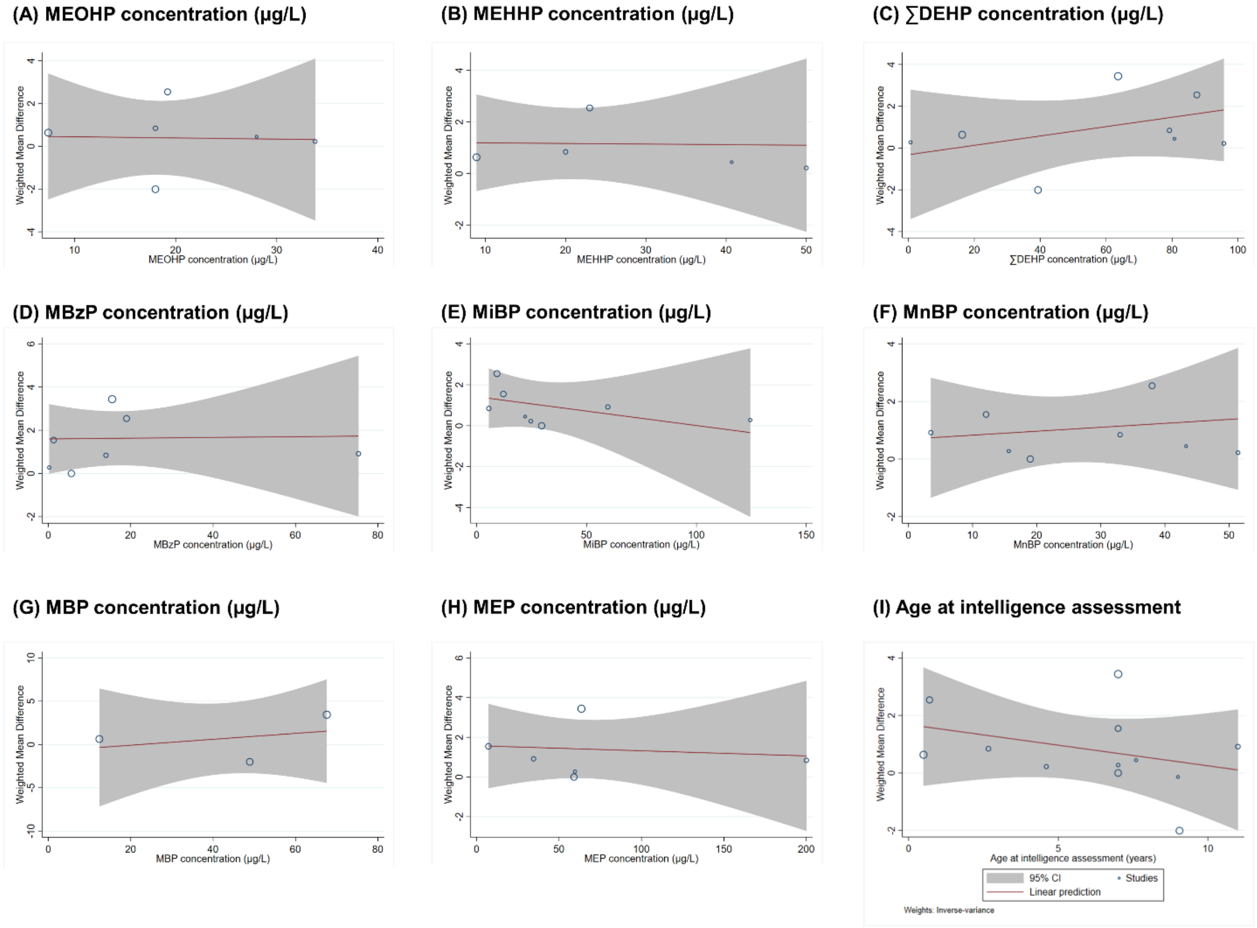

Figure 1. Meta-regression of the weighted mean difference and (A) MEOHP concentration (µg/L), (B) MEHHP concentration (µg/L), (C) ∑DEHP concentration (µmol/L), (D) MBzP concentration (µg/L), (E) MiBP concentration (µg/L), (F) MnBP concentration (µg/L), (G) MBP concentration (µg/L), and (H) MEP concentration (µg/L), and (I) age at intelligence assessment.

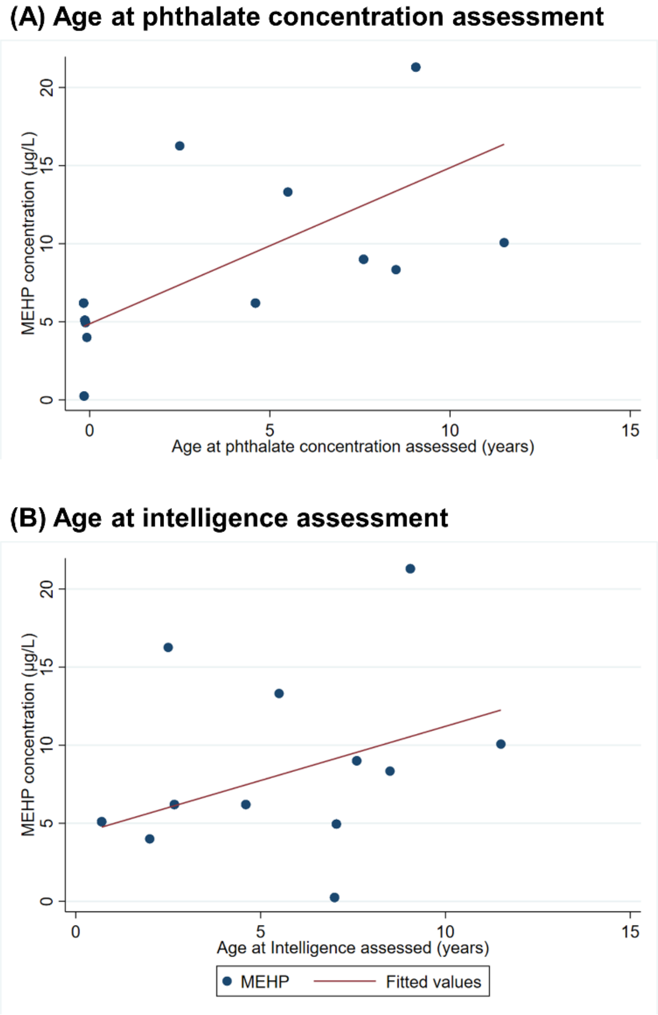

Figure 2. Correlation plots of MEHP concentration (µg/L) and (A) age at phthalate concentration assessment and (B) age at intelligence assessment.
